# 敏感驱动基因阳性非小细胞肺癌脑膜转移药物治疗的管理

**DOI:** 10.3779/j.issn.1009-3419.2020.102.18

**Published:** 2020-08-20

**Authors:** 志琴 陆, 婧 蔡, 治民 曾, 安文 刘

**Affiliations:** 1 330006 南昌，南昌大学第二附属医院肿瘤科 Department of Oncology, The Second Affiliated of Nanchang University, Nanchang 330006, China; 2 330006 南昌，江西省肿瘤临床转化重点实验室 Jiangxi Key Laboratory of Clinical Translational Cancer Research, Nanchang 330006, China

**Keywords:** 肺肿瘤, 脑膜转移, 驱动基因阳性, 药物治疗, Lung neoplasms, Leptomeningeal metastasis, Driver gene positive, Drug therapy

## Abstract

脑膜转移（leptomeningeal metastasis, LM）是晚期非小细胞肺癌（non-small cell lung cancer, NSCLC）严重并发症之一，虽然发生率不高，但临床症状表现严重，预后差。驱动基因阳性NSCLC较驱动基因阴性患者更容易发生LM。目前，LM的治疗方法主要包括分子靶向治疗、系统化疗、全脑放疗、鞘内化疗及免疫治疗等。尽管治疗手段很多，但LM的疗效仍不令人满意。本文就敏感驱动基因阳性NSCLC LM的药物治疗方面作一综述。

## Abstract (en)

Leptomeningeal metastasis (LM) is one of the serious complications of advanced non-small cell lung cancer (NSCLC), although the incidence is not high, the clinical symptoms are severe and the prognosis is poor. LM is prone to occur in patients with positive driver gene than negative. At present, the treatment of LM mainly includes molecular targeted therapy, systemic chemotherapy, whole brain radiotherapy, intrathecal chemotherapy and immunotherapy. Although there are many treatments, the efficacy of LM is still unsatisfactory. This article reviews the drug therapy of sensitive driver gene positive NSCLC LM.

## 1 前言

肺癌是全球发病率及死亡率最高的恶性肿瘤^[1]^，非小细胞肺癌（non-small cell lung cancer, NSCLC）占肺癌的85%^[2]^。脑膜转移（leptomeningeal metastasis, LM）是晚期NSCLC严重并发症之一，其发病率较低。NSCLC-LM的发生率约为3.8%，84%-96%为腺癌，约1/3的LM合并脑实质转移^[3]^。驱动基因阳性的NSCLC占58.8%，其中表皮生长因子受体（epidermal growth factor receptor, *EGFR*）基因突变占46.5%，间变性淋巴瘤激酶（anaplastic lymphoma kinase, *ALK*）基因融合占11.5%，*c-ros*癌基因1基因融合（*ROS-1*融合）占0.8%^[4]^，多项报道^[3, 5-8]^提示驱动基因阳性NSCLC较野生型更容易发生LM。LM患者预后差，若不治疗，生存期仅约6周-8周^[9]^，随着分子靶向治疗及免疫治疗的突破性进展，NSCLC患者的生存期进一步延长，LM的发病率也逐年上升^[8, 10]^。

## 2 临床表现与诊断

LM患者临床表现包括无症状性LM和有症状性LM。LM临床上往往临床表现比较严重，且与脑实质转移难以鉴别。LM的主要临床表现有：①脑实质受累及脑膜刺激表现：头痛、恶心呕吐、颈项强直、脑膜刺激征、认知障碍、癫痫发作和肢体活动障碍等；②颅神经受累表现：常见的受累脑神经有视神经、动眼神经、滑车神经、外展神经、面神经、听神经等，表现为视力下降、复视、面部麻木、味觉和听觉异常、吞咽和发音困难等；③颅内压增高表现（头痛、呕吐、视乳头水肿）和脑积水压迫脑组织引起的进行性脑功能障碍表现（智力障碍、步行障碍、尿失禁）等；④如同时伴有脊膜播散则还可出现脊髓和脊神经根刺激表现^[11]^。LM常呈多灶性，好发于颅脑基底池和小脑脑沟等部位^[7, 12]^。LM诊断主要基于神经系统症状、影像学证据和脑脊液资料的综合评价，脑脊液细胞学检查仍然是LM诊断的金标准。多次脑脊液检测可提高细胞学检查的阳性率，但若有明确的肿瘤病史、新发的神经系统症状及体征，再加上典型的颅脑磁共振成像（magnetic resonance imaging, MRI）表现如脑膜及神经根的强化、硬膜内结节等，也可诊断为脑膜转移^[13, 14]^。

## 3 LM分类

LM根据其驱动基因可以分为*EGFR*突变NSCLC脑膜转移、*ALK*基因融合NSCLC脑膜转移、*ROS-1*融合NSCLC脑膜转移。根据是否接受过治疗可以分为初治NSCLC脑膜转移、NSCLC治疗期间出现LM。根据是否有神经系统症状分为无症状性LM和有症状性LM。驱动基因不同、初治或复治、有无神经系统症状的患者，其治疗策略也不同。

## 4 治疗

LM治疗的目的是改善或稳定患者的神经系统症状、改善生活质量和提高生存率。一项研究^[15]^表明，LM经积极治疗可提高中位总生存时间（overall survival, OS）（中位OS 6.0个月*vs* 1.9个月；*P* < 0.001）。LM治疗后的中位生存时间为3个月-11个月^[16-18]^，1年生存率为19%^[19, 20]^。LM与脑实质转移稍有区别，LM预后更差，症状更重，目前没有单纯NSCLC脑膜转移的共识。对于无症状脑膜转移患者，针对其敏感驱动基因的不同、是否初治作进一步论述。对于有症状的LM患者，应在进行局部控制后脑膜转移症状稳定或消失后，按以上分类进一步论述。

### 4.1 驱动基因阳性NSCLC初治合并LM

22.6%的*EGFR*突变NSCLC在初次诊断为转移性NSCLC合并LM，22%的*ALK*基因融合NSCLC在初次诊断为转移性NSCLC合并LM^[3, 21]^。随着酪氨酸激酶抑制剂（tyrosine kinase inhibitors, TKIs）的广泛应用，驱动基因阳性NSCLC患者LM生存期明显延长^[3, 21]^。

脑脊液血药浓度是影响TKIs治疗LM的重要因素^[22-24]^，这些差异可能与血脑屏障（blood brain barrier, BBB）有关。不同TKIs治疗药物进入软脑膜间隙主要取决于理化特性和药理特性^[25]^，不同代TKIs或同一代不同药物在脑脊液的浓度不同，不同代TKIs或同一代不同药物在脑脊液的浓度不同^[22, 26]^。

#### 4.1.1 *EGFR*突变NSCLC初治合并LM

目前，TKIs治疗是*EGFR*突变NSCLC患者的一线治疗方案。对于具有*EGFR*突变NSCLC伴脑（膜）转移的患者，美国国立综合癌症网络（National Comprehensive Cancer Network, NCCN）、欧洲肿瘤内科学会（European Society for Medical Oncology, ESMO）等各大指南均推荐首选TKIs治疗。不同TKIs在脑脊液血药浓度不同，其治疗LM疗效也不同。

吉非替尼、厄洛替尼、埃可替尼是经典的第一代EGFR-TKIs。小鼠动物模型研究^[27]^表明厄洛替尼在脑脊液浓度高于吉非替尼。一项回顾性研究^[28]^表明，厄洛替尼比吉非替尼脑脊液浓度及渗透率更高，吉非替尼与厄洛替尼在脑脊液中的浓度[(8.2±4.3) nmol/L *vs* (66.9±39.0) nmol/L, *P*=0.000, 8]，脑渗透率[(1.13%±0.36%) *vs* (2.77%±0.45%), *P* < 0.000, 1）]，厄洛替尼治疗LM有效。另一项回顾性研究^[29]^显示厄洛替尼对NSCLC脑膜转移控制率比吉非替尼好，其证据是厄洛替尼的脑脊液细胞学转换率优于吉非替尼（64.3% *vs* 9.1%, *P*=0.012）。在一项Ⅰ期研究^[30]^中，大剂量吉非替尼（每天750 mg或1, 000 mg）致57%的NSCLC脑膜转移患者的神经系统症状得到改善，颅内中位无进展生存期（progression-free survival, PFS）为2.3个月（范围：1.6个月-4.0个月）；中位OS为3.5个月（范围：1.6个月-5.1个月）。上述多项研究提示：厄洛替尼在脑脊液浓度高于吉非替尼。因此，在仅一代EGFR-TKIs可及性的情况下，厄洛替尼优于吉非替尼、埃可替尼，推荐厄洛替尼每日150 mg治疗LM。

阿法替尼是第二代EGFR-TKI抑制剂，不可逆的ErbB家族[EGFR/ErbB1、人类表皮生长因子受体2（HER2; ErbB2）和ErbB4]阻滞剂。临床前研究显示阿法替尼在脑内浓度较低，在临床使用剂量中脑与血浆浓度比 < 0.36。在Ⅲ期临床试验LUX-Lung 3和LUX-Lung 6亚组分析结果显示，与化疗对比，对研究中包含的基线无症状脑转移瘤患者行亚组分析，均显示出无进展生存率的改善趋势，但差异无统计学意义^[31-33]^，故阿法替尼的中枢神经系统效应有待进一步研究。

奥希替尼是第三代EGFR-TKI，它选择性地抑制EGFR-TKI增敏和*EGFR* T790M抗性突变。基于有T790M突变的NSCLC设计的AURA系列（包括AURA扩展队列、AURA2、AURA3和AURA17）研究总共纳入22例T790M突变晚期NSCLC脑膜转移患者，结果LM的ORR高达55%，DCR达91%。LM中位PFS为11.1个月，中位OS为18.8个月（95%CI: 6.3-NC），12个月生存率为65%（95%CI: 40%-82%）^[34]^。临床前、Ⅰ期/Ⅱ期临床研究及AURA系列研究^[35]^表明，与一代、二代相比，奥希替尼有更高的脑渗透性，基于此开展了Ⅲ期FLAURA研究，研究纳入先前未治疗的*EGFR*敏感突变晚期NSCLC患者，结果表明：与第一代EGFR-TKIs相比，奥希替尼中位PFS达18.9个月，中位OS达38.6个月。基线合并脑转移（包括LM）亚组中，奥希替尼组的中位PFS为15.2个月，奥希替尼明显降低患者脑及脑膜转移进展风险^[36]^。因此，对于*EGFR*突变NSCLC脑膜转移初治患者，首选第三代的奥希替尼治疗，有T790M突变，其疗效更佳。

总之，第一代TKIs中，厄洛替尼优于吉非替尼和埃可替尼；另外，临床前及临床研究均提示奥希替尼在脑脊液中高于第一、二代TKIs，奥希替尼治疗*EGFR*突变NSCLC脑膜转移优于第一、二代。EGFR-TKIs治疗的总客观缓解率（objective response rate, ORR）高达30%-35%，总中位OS为3个月-12个月，大剂量优于标准剂量，三代TKIs优于一代、二代^[3, 22, 30, 37-43]^。脑脊液血药浓度是TKIs治疗LM疗效的重要因素。其他新型脑渗透率高的TKIs（如AZD3759和Tesevatinib）正在研发，有望提高脑及脑膜转移患者的疗效，期待进一步的临床研究数据^[44]^。

#### 4.1.2 *ALK*融合基因及*ROS-1*阳性NSCLC初治合并LM

*ALK*融合基因阳性NSCLC患者诊断时大约30%有中枢神经系统（central nervous system, CNS）转移，LM发生率为10.3%^[45]^，关于*ALK*阳性及*ROS-1*阳性LM治疗的研究很少。

克唑替尼是针对ALK/MET/ROS1的竞争性ATP抑制剂^[7, 22, 46]^。一代ALK-TKI克唑替尼对血脑屏障的穿透率不高，仅0.26%左右^[47]^，大剂量克唑替尼（250 mg, *bid*）联合鞘内注射甲氨喋呤*ALK*融合基因阳性NSCLC脑膜转移有效^[48]^。克唑替尼治疗LM，疗效差，联合鞘内化疗治疗LM可能有效。

色瑞替尼是第二代ALK/ROS1抑制剂，有报告^[49]^显示，色瑞替尼治疗ALK阳性NSCLC脑转移和软脑膜转移，颅脑控制时间达5个月。针对ALK阳性的有症状或进展期的NSCLC脑转移和/或脑膜转移的Ⅱ期ASCEND-7研究（NCT02336451），总共纳入156例脑及脑膜转移患者，其整体ORR达30%-60%，其中18例LM的ORR高达20%。一项针对*ALK*阳性NSCLC色瑞替尼对比化疗一线治疗的Ⅲ期ASCEND-4研究中，基线合并脑转移47例，色瑞替尼750 mg的ORR达44%（95%CI: 24.4%-65.1%），中位OS达10.7个月（95%CI: 8.1-16.4）色瑞替尼750 mg对颅内控制率高^[50]^。布加替尼是一种有效的二代ALK/ROS/EGFR抑制剂，在*ALK*阳性NSCLC患者中，颅内反应率达53%-67%，中位颅内PFS超过12个月。布加替尼在软脑膜转移中的活性仍有待确定，包括无症状LM的患者Ⅲ期ALTA-1L研究（NCT02737501）正在进行。阿来替尼是第二代ALK/RET抑制剂，具有高CNS渗透性。2019年ESMO更新的ALEX研究中，中位PFS高达38.6个月，4年OS率达64.5%，对于基线合并CNS转移患者，阿来替尼和克唑替尼组的中位PFS分别为25.4个月和7.4个月（HR=0.37, 95%CI: 0.23-0.58），对于基线有CNS转移的患者，阿来替尼组降低了40%的死亡风险（HR=0.6, 95%CI: 0.34-1.05）。对于有*ALK*融合阳性NSCLC合并CNS转移（包括脑膜）患者，二代优于一代，阿来替尼优于色瑞替尼、布加替尼、克唑替尼。

劳拉替尼是第三代ALK/ROS1抑制剂，动物模型劳拉替尼血脑屏障渗透率达31%-96%。Ⅱ期临床研究结果显示劳拉替尼治疗ALK阳性NSCLC的颅内客观应答率高达73%^[51]^，Gafer等^[52]^报道了2例劳拉替尼治疗*ALK*基因融合NSCLC脑及脑膜转移的患者，提示劳拉替尼治疗快速进展性脑和LM患者有效。

总之，对于有*ALK*融合阳性NSCLC伴脑及脑膜转移患者，阿来替尼优于劳拉替尼、色瑞替尼，劳拉替尼优于色瑞替尼，二代ALK-TKIs颅内PFS均优于克唑替尼^[53, 54]^。其他新型ALK-TKIs entrectinib和ensartinib（NCT02568267、NCT01625234）对LM疗效的研究正在进行当中。

### 4.2 驱动基因阳性NSCLC TKIs治疗后出现LM的管理

大约9%-10%具有*EGFR*基因突变的NSCLC患者在TKIs治疗后产生耐药而出现LM^[3]^，其中，*EGFR*中含有L858R突变的患者比*EGFR*外显子19缺失的患者更有可能发生LM（10.7% *vs* 3.4%, *P*=0.006）^[15]^。LM通常是NSCLC的晚期事件，52%（范围：9%-76%）有神经系统症状^[55]^。60.9%患者在诊断LM之前至少接受了一种EGFR-TKIs方案治疗^[3]^，TKIs治疗后出现LM的治疗是临床上较为棘手问题之一，尚缺乏最佳治疗策略。

TKIs治疗耐药后出现LM，神经系统症状较重，可考虑联合局部和/或全身治疗。然而，是TKIs剂量加强或更换TKIs还是TKIs联合抗血管生成，抑或是TKIs联合放疗或TKIs联合鞘内化疗，目前尚无共识。如何最佳运用全身系统联合局部治疗的多学科思维模式，让LM患者最大获益，需要大量临床数据。结合颅内外肿瘤控制情况，LM的治疗策略也不同。

#### 4.2.1 颅内进展

TKIs治疗后出现LM，并且颅外病灶控制情况良好，以中枢及脑膜转移为主，可考虑以下治疗策略；以下主要针对*EGFR*突变NSCLC TKIs治疗后出现LM的治疗模式的选择。

##### 4.2.1.1 TKIs剂量加强或更换TKIs

TKIs耐药后出现包括脑膜转移在内的中枢神经系统进展是临床的常见问题。倘若颅外病灶控制良好的情况下，中枢也许在高剂量及换代情况下仍有效，可考虑TKIs剂量加强或者更换奥希替尼治疗。Flippot教授^[56]^的一项多中心回顾性研究中，总共纳入92例患者，LM诊断后的中位OS为6.1个月（95%CI: 4.2-7.6），87例TKIs治疗失败后再次接受TKIs治疗的患者（*n*=50）的中位LM OS为7.6个月（95%CI: 5.7-10.9），而未接受进一步治疗的患者为4.2个月（95%CI: 1.6-6.7），60%再次接受TKIs挑战的患者临床受益，在阿法替尼或吉非替尼之后用厄洛替尼治疗的患者中有11/20（55%）报告了临床获益，得出结论TKIs耐药后NSCLC-LM再挑战TKIs，包括剂量加强，临床可获益。一项35例大剂量厄洛替尼治疗标准剂量EGFR-TKIs治疗失败的难治性LM，12例接受了大剂量的厄洛替尼（每2天200 mg或300 mg，或每3天300 mg或450 mg，或每4天600 mg）治疗，23例仅接受了标准剂量的EGFR-TKIs。在接受大剂量厄洛替尼的患者中，3例（30%）证实了影像学有效。大剂量厄洛替尼患者诊断LM后的中位OS为6.2个月（95%CI: 2.5-8.5），没有≥3级毒性、间质性肺疾病或治疗相关的死亡，得出结论大剂量厄洛替尼是标准剂量EGFR-TKI失效后的难治性LM的潜在治疗选择^[57]^。一项9例回顾性分析脉冲式大剂量厄洛替尼（每周1, 500 mg）治疗*EGFR*突变型NSCLC的脑及LM，颅脑影像学反应部分为67%（6/9，包括2例LM），中位颅脑PFS达2.7个月（范围：0.8个月-14.5个月），中位OS为12个月^[39]^。厄洛替尼可以克服吉非替尼治疗过程中出现的LM^[58]^。因此，对于TKIs治疗期间出现的LM，可选择TKIs剂量加强（包括脉冲式给药或大剂量给药）或者更换奥希替尼治疗。

##### 4.2.1.2 TKIs联合放疗

对于NSCLC的LM患者，TKIs耐药后，神经系统症状明显，全脑放疗是否是一种有益的治疗？一项小样本研究^[15]^提示TKIs联合全脑放疗的患者的生存时间明显长于未接受全脑放疗（whole brain radiation therapy, WBRT）的患者（6.0个月*vs* 3.9个月，*P*=0.048），全脑放疗良好的预后因素。Liao等^[3]^的一项关于184例LM治疗的回顾性分析，128例（60.4%）患者在诊断为LM后接受全脑放疗，中位OS为4.5个月（95%CI: 3.5-7.3）。多因素分析表明全脑放疗是长期生存的独立预测因素。Yan等^[59]^回顾性研究了51例*EGFR*突变NSCLC-LM患者，全脑放疗组26例与非全脑放疗组25例的颅内PFS没有差异（中位3.9个月*vs* 2.8个月；HR=0.506，*P*=0.052），得出结论*EGFR*突变阳性的NSCLC-LM，全脑放疗在统计学上并未改善颅内治疗反应和生存率。ESCO推荐全脑放疗用于广泛结节状或有症状的线性LM，对于局限性LM，特别是有症状的病变，应考虑局灶性放疗^[7]^。

##### 4.2.1.3 TKIs联合抗血管生成

贝伐珠单抗是一种抗血管内皮生长因子（vascular endothelial growth factor, VEGF）的单克隆抗体。动物研究和尸检标本表明VEGF在LM中起着重要作用^[60]^。Ariyasu等^[61]^报道的2例厄洛替尼联合贝伐单抗治疗*EGFR*突变NSCLC-LM有效。2019年美国临床肿瘤学会（American Society of Clinical Oncology, ASCO）-Abstract No9086奥希替尼联合贝伐珠单抗作为*EGFR*突变阳性肺癌患者初始治疗的Ⅰ期/Ⅱ期研究，其总缓解率为80%，中位PFS为18.4个月，1年PFS率为76%，1年OS率为91%，奥希替尼联合贝伐珠单抗治疗是安全的。最近的研究表明，与单独使用EGFR-TKIs相比，EGFR-TKIs联合贝伐单抗延长了*EGFR*突变NSCLC和多发性脑转移患者的PFS和OS^[62, 63]^。然而，奥希替尼联合贝伐珠单抗治疗*EGFR*突变NSCLC脑膜转移有待进一步大样本临床研究进一步验证。因此，TKIs联合贝伐珠单抗是潜在的选择。

##### 4.2.1.4 TKIs联合鞘内化疗

甲氨蝶呤（methotrexate, MTX）、阿糖胞苷（cytarabine, Ara C）及硫代三乙烯磷胺（triethylene thiophosphoramide, thio TEPA）是LM患者鞘内治疗的三种常用药物^[7, 64]^。鞘内联合化疗也并不优于单一药物治疗。Ommaya囊内给药优于腰椎穿刺给药^[64, 65]^。潘振宇教授^[66]^一项关于鞘内注射培美曲塞治疗NSCLC复发性LM的安全性及可行性的前瞻性单臂Ⅰ期临床试验（NCT03101579），疾病控制率高达54%（7/13），提示培美曲塞10 mg鞘内注射治疗LM疗效好，毒性可控。鞘内注射贝伐珠单抗治疗LM的动物模型为贝伐珠单抗鞘内注射治疗LM提供了安全性数据，以允许在治疗难治性LM的人类中进行Ⅰ期/Ⅱ期研究。ESMO专家共识推荐鞘内化疗应该推荐用于大部分结节脑脊液阳性结节型或线型（IA/C型）、CSF中肿瘤细胞负荷较大的LM患者，Ommaya囊内给药优于腰椎穿刺给药，培美曲塞是新的选择。

#### 4.2.2 颅内合并颅外进展

颅内颅外均有进展，全身治疗是必然的，可同时兼顾全身及颅内中枢病灶的治疗。因预期生存期短，可选择更换TKIs、系统化疗、联合化疗或免疫治疗。

##### 4.2.2.1 更换TKIs

颅内颅外均进展，可考虑行二次活检。若组织或血液二代测序提示有*EGFR*突变阳性，更换第三代奥希替尼治疗^[36]^；若*ALK*融合阳性或*ROS-1*阳性可更换阿来替尼或者劳拉替尼^[51, 52]^。

##### 4.2.2.2 抗血管生成联合化疗

全身化疗是TKI耐药后产生中枢转移的生存预后良好因素，对颅内合并颅外进展，化疗联合抗血管生成可能有效。Soria等^[67]^发表了一篇关于贝伐珠单抗联合一线含铂为基础治疗晚期NSCLC脑膜转移的随机临床Ⅱ期/Ⅲ期试验的*meta*分析和系统评价，总共纳入2, 194例患者（1, 313例贝伐珠单抗组、881例对照组），与单纯化疗组相比，加入贝伐珠单抗组的OS明显延长（HR=0.90, 95%CI: 0.81-0.99, *P*=0.03）和PFS（HR=0.72, 95%CI: 0.66-0.79, *P* < 0.001）。一项50例NSCLC脑膜转移的研究分析显示，诊断LM后进行系统化疗与未进行系统治疗比较，中位OS差异明显（11.5个月*vs* 1.4个月，*P* < 0.000, 1）。系统化疗是生存预后良好因素。颅内颅外均有进展，可考虑全身化疗，推荐培美曲塞铂联合贝伐珠单抗方案治疗。

##### 4.2.2.3 TKIs联合化疗

杨海虹等^[68]^回顾性分析了6例培美曲塞/顺铂化疗期间间插厄洛替尼治疗吉非替尼耐药后*EGFR*突变NSCLC脑膜转移患者，给药方式为培美曲塞500 mg/m^2^，d1，顺铂30 mg d1-d2厄洛替尼150 mg，d3-d20，21 d为1个周期，至少接受4个周期的化疗，4例患者表现出良好的反应（完全和部分反应），2例患者有颅内肿瘤方面的稳定疾病，所有患者的表现和转移相关的神经症状都得到了改善，OS为8个月-15个月（中位9个月），最常见不良反应为皮疹、口腔黏膜炎或甲沟炎，总体耐受性良好。对*EGFR*突变NSCLC脑膜转移，厄洛替尼联合培美曲塞/顺铂可获得良好的局部应答率。一项培美曲塞治疗LM的回顾性分析^[69]^提示，LM后使用培美曲塞的患者与不使用的中位OS（13.7个月*vs* 4.0个月，*P*=0.008），多变量分析显示LM后使用培美曲塞与生存有关（HR=3.1, 95%CI: 5-6.3, *P*=0.002）。颅外进展，可考虑联合培美曲塞单药或含培美曲塞化疗。

##### 4.2.2.4 免疫治疗

目前，免疫检查点抑制剂（immune checkpoint inhibitors, ICIs）治疗是局部晚期/转移性NSCLC的一线标准治疗^[70, 71]^。由于多数的临床试验基本排除了LM的患者，所以ICIs治疗LM数据不多。个案报道提示Nivolumab对NSCLC CNS转移患者可能具有颅内活性和良好的安全性^[72, 73]^，Ⅱ期研究Pembrolizumab对LM的疗效正在进行（NCT03091478）。Hendriks等^[74]^回顾性分析了19例ICIs治疗NSCLC脑膜转移患者，其中3例有*EGFR*突变，1例*ALK*融合基因阳性，用Pembrolizumab/Nivolumab治疗，ICIs中位PFS为2.0个月（95%CI: 1.8-2.2），NCCN预后良好组的6个月PFS率为40%，中位OS为3.7个月（95%CI: 0.9-6.6），6个月OS率为36.8%，12个月OS率为21.1%，NCCN LM预后良好组可从ICIs治疗中临床获益。ICIs治疗起效相对缓慢，但一旦有效，维持时间相对较长，更多ICIs治疗LM的疗效有待进一步研究。

### 4.3 非TKIs治疗后复发出现的LM

非TKIs治疗后复发出现的LM指无TKIs治疗史的LM患者，如初始行手术治疗和/或放疗和或化疗后出现脑膜复发进展者。复发后将进行基因检测，依据基因检测结果进行后续治疗。对于非TKIs治疗复发出现的LM，无症状或者临床症状较轻的患者，可根据其相应驱动基因，选择相应药物，如*EGFR*突变阳性的LM患者，首选第三代的奥希替尼靶向治疗^[36]^；有*ALK*融合/*ROS-1*阳性，可考虑行阿来替尼或劳拉替尼靶向治疗^[51, 52]^。对于临床症状较重的患者，应进行局部控制LM相关症状，TKIs治疗的同时联合全脑放疗、鞘内化疗或抗血管生成治疗。对于无驱动基因突变的患者，积极对症治疗的同时根据其Karnofsky体能状态（Karnofsky performance status, KPS）评分可选择培美曲赛化疗联合抗血管生成药物贝伐珠单抗^[67]^或免疫治疗^[72-74]^。

## 5 预后

诊断明确后对LM进行预后评估，及时发现预后良好的亚群并及时给予准确的临床干预。目前NCCN指南指出RPA、molGPA是预测实体瘤脑/中枢系统转移的经典预测模型。RPA、molGPA主要基于KPS评分、颅外转移（extracranial metastasis, ECM）、基因状态和症状。NCCN预后良好包括EGFR/ALK阳性、年龄 < 50岁、KPS≥90分、无ECM，预后差包括EGFR/ALK阴性、年龄 > 60岁、KPS < 70分、有ECM。近期吴一龙教授根据基因状态、KPS、ECM将肺癌LM患者分为高风险、中风险和低风险，高风险包括*EGFR*/*ALK*突变、KPS < 60分、有ECM，低风险包括*EGFR*/*ALK*阳性、KPS≥80分和无ECM，低风险OS较高风险患者明显延长。LM属于中枢系统转移，但有别于实质转移，因此LM的预后适用上述模型，基因状态、KPS、ECM是LM的预后因子^[3, 6, 7, 38, 75-77]^（表 1）。

**1:** 靶向药物的血浆/脑脊液浓度及脑脊液渗透率 Plasma/cerebrospinal fluid concentration and cerebrospinal fluid permeability of targeted drugs

Drug	Plasma concentration	CSF concentration	CSF permeability
Gefitinib	(729±260) nmol/L	(8.2±4.3) nmol/L	1.13%±0.36%^[28]^
Erlotinib	(717.7±459) nmol/L	(66.9±39.0) nmol/L	2.77%±0.45%^[27-29]^
High dose erlotinib	11.3 nmol/L	130 nmol/L	1.2%^[57]^
Afatinib	66.7 ng/mL	0.464 ng/mL	< 1%^[31-33]^
Osimertinib	13 nmol/L	7.5 nmol/L	2.5%-16%^[41]^
AZD3759	0.2 nmol/L	25.2 nmol/L	100%^[44]^
Crizotinb	237 ng/mL	0.616 ng/mL	0.26%^[47]^
Alactinib	3.12 nmol/L	2.69 nmol/L	86%^[26, 77]^
Ceritinib	Unreported	Unreported	15%^[77]^
Brigatinib	0.62 nmol/L^[77]^	Unreported	Unreported
Lorlatinib	< 0.07 nmol/L	2.64 ng/mL-125 ng/mL	20%-30%^[51]^
CSF: central nervous system.			

## 6 小结

LM是NSCLC严重、致死性的并发症，随着TKIs的广泛使用，驱动基因阳性NSCLC脑膜转移患者的总生存明显延长。对于驱动基因阳性NSCLC脑膜转移以及TKIs治疗耐药后出现LM进展的患者，皆应进行详细的预后评估，根据基因突变情况、KPS评分、有无颅外转移以及既往治疗情况制定合理的综合治疗方案，以最大限度地提高患者生活质量，改善患者预后。
